# Supplementary material for: Antitumor Effect of Berberine Analogs in a Canine Mammary Tumor Cell Line and in Zebrafish Reporters via Wnt/β-Catenin and Hippo Pathways
Source: Biomedicines. 2023 Dec 15;11(12):3317. doi: 10.3390/biomedicines11123317 (PMC10741123; doi:10.3390/biomedicines11123317)
Supplement: Supplementary file 1 [file biomedicines-11-03317-s001.zip › biomedicines-2735149-supplementary.pdf]

biomedicines

Supplementary Materials

Table S1. Half maximal inhibitory concentration (IC50) and standard deviation (SD) of different compounds after 24 hours of treatment of CF33 cells.

Drug	IC50 ± SD (µM)
Berberine (BBR)	>40
NAX012	8.9 ± 2.7
NAX014	11.0 ± 1.2
NAX035	10.0 ± 1.8
NAX053	6.2 ± 1.6
NAX057	5.9 ± 3.3
NAX060	6.8 ± 0.9
Berberrubine (BRR)	>40
NAX085	>40
NAX118	>40

Table S2. Different drugs toxicity effects (LD50) and standard deviation (SD) on zebrafish larvae treated for 24 hours.

Drug	LD50 ± SD (µM)
Berberine (BBR)	200 ± 1.40
NAX012	100 ± 0.73
NAX014	100 ± 0.35
NAX035	20 ± 0.42
NAX053	10 ± 0.35
NAX057	10 ± 0.41
NAX060	20 ± 0.64
Berberrubine (BRR)	200 ± 1.24
NAX085	200 ± 1.33
NAX118	200 ± 1.17
